# Cesarean Delivery on Maternal Request among Patients Undergoing Cesarean Section in a Tertiary Care Hospital: A Descriptive Crosssectional Study

**DOI:** 10.31729/jnma.5691

**Published:** 2021-05-31

**Authors:** Dipty Shrestha, Rachana Saha, Shilpi Mahato

**Affiliations:** 1 Department of Obstetrics and Gynaecology, Kathmandu Medical College and Teaching Hospital, Sinamangal, Kathmandu, Nepal

**Keywords:** *anxiety*, *cesarean section*, *labor pain*

## Abstract

**Introduction:**

Caesarean delivery on maternal request in absence of any maternal and fetal indications and has become a concerning issue among obstetricians. It seems to be one of the contributory factors of increased cesarean rate all over the world. This study aims to find out the prevalence of cesarean delivery on maternal request among cesarean deliveries in a tertiary care hospital.

**Methods:**

This descriptive cross-sectional study was conducted from November 1st 2019 to February 1st 2020 among women undergoing cesarean section in a tertiary care hospital. The ethical clearance was taken from the Institutional Review Committee of Kathmandu Medical College (reference number: 201120192). Convenient sampling was used. Statistical Package for Social Sciences version 20.0 was used for analysis. Point estimate at 95% Confidence Interval was calculated along with frequency and proportion for binary data.

**Results:**

Out of 386 cesarean sections, maternal request was the indication in 72 (18.65%) (95% Confidence Interval = 14.76-22.54) mothers. Among the 72, 38 (52.7%) chose cesarean section for fear of labor pain, 14 (19.4%) for date-of-birth selection, 10 (13.8%) for anxiety of labor pain, because of cord around the neck in four (5.5%), male baby in three (4.1%), to avoid pelvic trauma in two (2.7%), and to go abroad in one (1.3%).

**Conclusions:**

Our study showed a prevalence of cesarean delivery on maternal request higher than other national studies but was similar to the global prevalence. The commonest reasons were fear of labor followed by date-of-birth selection.

## INTRODUCTION

Caesarean Delivery on Maternal Request (CDMR) is defined as a primary cesarean delivery on maternal request in the absence of any maternal or fetal indications.^1^ In Nepal CDMR is gaining popularity day by day and is in increasing trend.

The incidence of CDMR and its contribution to the overall increase in the cesarean delivery rate is not well known, but it is estimated that 2.5% of all births in the United States are cesarean delivery on maternal request.^1^ Incidence of CDMR reached up to 18% of total cesarean delivery worldwide.^2^ In a study in Nepal, the CDMR was found to be 1.25%.^3^ With the anticipated fear and anxiety of labor and its pains, pregnant mothers especially primigravidas approaches the obstetricians for elective cesarean sections.^4^

This study aims to find the prevalence of cesarean delivery on maternal request among cesarean deliveries in a tertiary care center.

## METHODS

This was a descriptive cross-sectional study conducted at the Kathmandu Medical College and Teaching Hospital (KMCTH) from November 1st, 2019 to February 1st, 2020 where women undergoing cesarean section on maternal request were taken into account to identify the various reasons for CDMR. Ethical clearance (reference no. 201120192) was taken from the Institutional Review Committee of the KMCTH. The study included all the pregnant women undergoing cesarean section (CS) after 37 weeks of gestation. The exclusion criteria included pregnant women less than 36 weeks of gestation and women undergoing cesarean section for obstetric emergencies. Each participant, before their enrollment, was explained about the study and its aim, and informed consent was taken. Convenience sampling was done and the sample size was calculated using the formula,


$$\begin{array}{ll} \text{n} & \text{= }{\text{Z}}^{\text{2}}\times \text{p}\times \text{q}/{\text{e}}^{\text{2}}\\ & \text{= }{\left(1.96\right)}^{\text{2}}\times 0.5\times (1-0.5)/{(\text{0.05)}}^{\text{2}}\\ & \text{= }384.16 \end{array}$$

Where,

n = minimum required sample size
Z = 1.96 at 95% Confidence Interval (CI)
p = prevalence taken as 50% for maximum sample size
q = 1-p
e = margin of error, 5%

The minimum required sample size was 384.16. But we included 386 cesarean deliveries in the study. Among all the cases undergoing a cesarean section, each CMDR case was interviewed regarding the reason for choosing CDMR using a well-structured questionnaire. The questionnaire included the demographic profile, obstetric history, family history, reasons for CDMR which included fear of labor pain, the anxiety of labor pain, date of birth selection, male baby, avoid pelvic floor trauma, unaware of epidural analgesia, cord around the neck, superstitious belief and others.

All the data were entered in Statistical Package for Social Sciences (SPSS) version 20.0 and analyzed. Data were analyzed using descriptive statistics and frequency and proportion were calculated for binary data. Point estimate at 95% CI was calculated along with frequency and proportion for binary data.

## RESULTS

**Figure 1.**
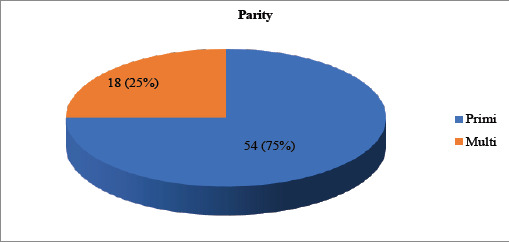
Parity index of patients requesting a cesarean delivery.

Out of the total patients undergoing cesarean section, CDMR was the indication in 72 (14.76-22.54 at 95% CI) cases. Among the 72 CDMR cases, 54 (75%) of them were primigravida and 18 (25%) were multigravida (Figure 1).

Only one (1.38%) case was in the group of >40 weeks of gestation and 71 (98.6%) of the cases were between 37 to 40 weeks of gestation (Table 1).

**Table 1:** Period of gestation of patients requesting a cesarean delivery.

Period of gestation	n (%)
37 - 40 weeks	71 (98.6)
>40 weeks	1 (1.38)
Total	72 (100)

**Figure 2.**
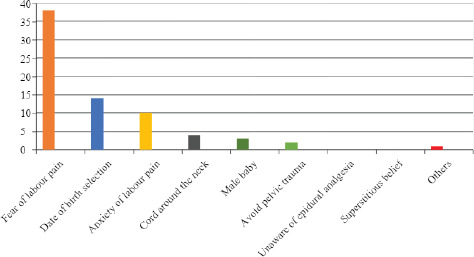
Reasons for requesting a cesarean delivery.

In 38 (52.7%) cases, the reason for CDMR was fear of labor followed by the date of birth selection in 14 (19.4%) cases. Other reasons for choosing CDMR were anxiety of labor pain 10 (13.8%), cord around the neck in four (5.5%), male baby in three (4.1%), avoid pelvic floor trauma in two (2.7%), and others like going abroad in one (1.3%) (Figure 2).

In total four babies were admitted to the neonatal intensive care unit (NICU), two (50%) for meconium-stained liquor, one (25%) for grunting, and one (25%) for mother being diabetic (Table 2).

**Table 2:** Reasons for NICU admission.

**NICU Admission**	**n (%)**
Meconium	2 (50)
Grunting	1 (25)
Mother Diabetes	1 (25)
Total	4 (100)

## DISCUSSION

CDMR in an increasing trend is seen as the option for delivery among most of the women today and what could be the reasons for CDMR is a major concern that has to be looked into. First, tocophobia (fear of childbirth) may be the most common reason for the increasing rate of CDMR.^5^

In 2006, Tang, et al. reported CS delivery increased from 18% in 1992 to 40% in 2000 in Urban China.^6^ In a study by Anwar E Ahmed and Rouzait S. Mohammad in Saudi Arabia the prevalence of CDMR was found to be 13.7%.^7^ A study by Yajun Liu, et al. revealed that the overall rate of CS in Mainland China was 54.90% and the most common indication for CS was maternal request (28.43%). CDMR accounted for 15.53% of all deliveries in Mainland China.^8^ A study by Chong, et al. has reported similar results, wherein 50% of their sample had their relatives or friends requesting a CS.^9^ CDMR was 2.9 times higher among pregnant women with a family history of CS than among those without a family history of CS.^7^ Pregnant women who received education or medical information about this issue were 13.7 times more likely to request a CS in the absence of medical indications than those who did not receive education or medical information.^7^ In a study by Fatemah, et al in total 20.8% of cesarean deliveries were CDMR, and the rate of CDMR in a private hospital and public hospitals was 87 (42.44%) and 18 (6.08%) respectively.^10^

In our study, most of the women demanding CDMR were primigravida that is 75%, and 25% were multigravida In a study by Ahmed, et al. in Saudi Arabia the mean age of the women was 31.3 years with an age range of 16-45 years.^7^ A study by Polat Dursen, et al. revealed that the mean age of the study population was 32+/-10.2 years, 47 of the women indicated that CD could be performed by the maternal request without medical indication while 28% and 25% of women were against this and had no idea respectively.^11^ In a study by Liat Lerver, et al, women in the CDMR were significantly older than those in the VD group with a median age at delivery of 33.4 versus 30, 3 years.^12^

In this study of mine, the commonest indication for CDMR was found to be fear of labor pain that accounted for 52.7% followed by the date of birth selection which was the choice of CDMR in 19.4% of the women. Other indications included anxiety of labor pain (13.8%), cord around the neck (5.5%), male baby (4.1%), avoid pelvic floor trauma (2.7%), and others like going abroad (1.3%). Similar to our study done by Anwar E Ahmed and Rouzait S. Mohammad in Saudi Arabia, the most common motives for demanding a CS in the absence of medical indications were to avoid labor or possible complications from vaginal birth (60%), followed by fear of pain on vaginal delivery (46%), concerns about unsuccessful vaginal birth after a CS (30%) and a previous traumatic delivery(26%).^7^ In a study by Yajun Liu, et al. the four leading indications for CS were maternal request (28.43%), cephalopelvic disproportion (14.08%), fetal distress (12.46%), and previous CS delivery (10.25%).^8^ It was also observed that male infants were more likely than female infants to be delivered by CS.^8^ On contradictory a survey conducted in the U.S showed that the leading four indications for CS were prolonged labor (dystocia), previous CS delivery, breech presentation, and fetal distress.^13^ It has been estimated that 6-10% of all pregnant women have a severe fear of childbirth.^14^ A survey in 2012 by Pawelec, et al. reported that 12% of CS requests by mothers were because of fear of labor pain and this had increased from a rate of 2%.^15^ Mancuso, et al. reported that the reason for this increasing CDMR trend is complex, varies from one region to another, and is influenced by local sociocultural and healthcare backgrounds.^16^ In the study by Liat Lerver, et al. the reasons for CDMR in the order of descending frequency were a concern for pain (21.9%), concern for risk to their own and fetal health (20.4%) and (16.5%) respectively, emotional aspects (10%), maternal and health condition (9.1%), traumatic obstetric history (9.1%), and other (12.1%).^12^

Ours was a single-centered study with a limited sample size and the participants were enrolled through the convenience sampling technique. Our findings, therefore, may not be generalized. A larger multicentered study with a more diverse population needs to be conducted. Our design restricted us from finding associations between variables. Therefore, analytical studies need to be done to establish the association of various reasons for which mothers request a cesarean delivery with the occurrence of CDMR.

## CONCLUSIONS

The prevalence of CDMR was higher than the findings from other national studies. But it was similar to the global prevalence. In our study, the commonest reason for CDMR was fear of labor pain followed by the purpose of date of birth selection. One of the contributory factors of the increasing rate of cesarean section is seen to be CDMR but a larger scale study is needed to come to a definite conclusion.
